# Evaluation of Hepatic Toxicity after Repeated Stereotactic Body Radiation Therapy for Recurrent Hepatocellular Carcinoma using Deformable Image Registration

**DOI:** 10.1038/s41598-018-34676-1

**Published:** 2018-11-01

**Authors:** Sumin Lee, Hojin Kim, Yunseo Ji, Byungchul Cho, Su Ssan Kim, Jinhong Jung, Jungwon Kwak, Jin-hong Park, Sang-wook Lee, Jong Hoon Kim, Sang Min Yoon

**Affiliations:** Department of Radiation Oncology, Asan Medical Center, University of Ulsan College of Medicine, Seoul, 05505 Republic of Korea

## Abstract

This study aimed to evaluate hepatic toxicity after repeated stereotactic body radiation therapy (SBRT) for recurrent hepatocellular carcinoma (HCC) using deformable image registration (DIR). Between January 2007 and December 2015, 85 patients who underwent two sessions of SBRT for HCC treatment were retrospectively analyzed. A DIR technique was used to calculate the cumulative dose of the first and second SBRT to the normal liver by matching two computed tomography simulation images. The Dice similarity coefficient (DSC) index was calculated to evaluate DIR accuracy. Before the first and second SBRT, 6 (7.1%) and 12 (14.1%) patients were Child-Pugh class B, respectively. Median tumor size was 1.7 cm before both SBRT treatments. Mean DSC index value was 0.93, being >0.9 in 79 (92.9%) registrations. Median cumulative mean liver dose (MLD) was 9.3 Gy (interquartile range, 7.6–11.7). Radiation-induced liver disease developed in three patients, and two of them, with Child-Pugh class B, experienced irreversible liver function deterioration following the second SBRT. The DIR method provided reliable information regarding cumulative doses to the liver. In patients with Child-Pugh class A liver function, repeated SBRT for small recurrent HCC could be safely performed with acceptable hepatic toxicity.

## Introduction

For early stage hepatocellular carcinoma (HCC), curative treatments, such as liver transplantation, surgical resection, and percutaneous ablation therapies, can be performed with excellent 5-year overall survival rates of up to 70%^1–3^. However, various clinical conditions present major restrictions in the application of standard curative treatments. Liver transplantation is limited to a small number of patients because of its strict indication and lack of donors. Surgical resection can only be performed in patients with sufficient liver function and a resectable tumor location^4,5^. Radiofrequency ablation (RFA) has a high rate of local control with a chance of cure^6,7^. However, tumors located near the liver surface, great vessels, gallbladder, and diaphragm, are significant obstacles in using RFA^8^. Historically, transarterial chemoembolization (TACE) is often used for patients suitable for curative treatments based on the positive results of the randomized trials^9,10^. However, local control after TACE is not as satisfactory as that achieved with curative therapies^11^.

Radiotherapy for HCC was not an attractive option in the past because the liver is known as a radiation-sensitive organ. However, recent advances in radiotherapy techniques have enabled safe delivery of high doses of radiation to focal liver lesions^12^. Many prospective and retrospective studies have reported that stereotactic body radiation therapy (SBRT) achieved excellent local control rates of 85–100% with acceptable toxicity^13–20^.

One of the major early stage HCC failure patterns after prior treatments, including surgery, RFA, or SBRT is another intrahepatic recurrence. Because of this tendency, HCC patients often require repeated locoregional treatments. If the recurrent lesion was also unsuitable for curative treatments after prior SBRT, the physician would have to consider another SBRT for the new recurrent HCC. In this case, hepatic toxicity or other late toxicities should be considered before the decision to administer another SBRT. In addition, the cumulative radiation dose to the liver after the first and second SBRT is required to predict hepatic toxicity. However, few studies have analyzed the safety of repeated SBRT and provided a dosimetric guideline. Therefore, in this study, we aimed to evaluate the safety of repeated SBRT for patients with recurrent HCC and to investigate the relationship between dose-volume parameters and the risk of hepatic toxicities using deformable image registration (DIR).

## Results

### Characteristics of patients and SBRT

Overall, 85 patients with a total of 170 SBRT sessions were analyzed. The characteristics of patients and each SBRT session are summarized in Table 1. Median age at the first SBRT was 64 years. Median tumor size was 1.7 cm in both the first and second SBRT sessions (interquartile range [IQR], 1.5–2.2 and 1.4–2.2, respectively). Tumors larger than 3 cm at the longest diameter in each SBRT session were only 8.2% and 5.9%, respectively. Only four patients received SBRT as the first-line treatment, and 13 (15.3%) patients underwent surgery before the first SBRT. Median interval between the two SBRT sessions was 15 months (IQR, 7–24). During this interval, 38 (44.7%) patients received other locoregional treatments with a median number of 2 (IQR, 1–3). The most commonly used dose-fractionation scheme was 45 Gy in three fractions. There was no significant difference in tumor size, the gross tumor volume (GTV), and planning target volume (PTV) between the two SBRT sessions. The mean liver dose (MLD) was 5.5 Gy and 5.0 Gy for the first and second SBRT, respectively.

**Table 1:** Characteristics of the patients and each session of SBRT.

N = 85	First SBRT	Second SBRT
Age (years)		
Median (IQR)	64 (56–70)	65 (57–71)
Sex		
Male	67 (78.8%)	
Female	18 (21.2%)	
Etiology		
Hepatitis B virus	60 (70.6%)	
Hepatitis C virus	18 (21.2%)	
Non-B, Non-C	7 (8.2%)	
ECOG performance status		
0–1	82 (96.5%)	83 (97.6%)
2	3 (3.5%)	2 (2.4%)
Previous local treatment^*^		
No	4 (4.7%)	47 (55.3%)
Yes	81 (95.3%)	38 (44.7%)
Surgery	13 (15.3%)	0 (0.0%)
Sessions of treatment		
Median (IQR)	3 (2–6)	2 (1–3)
Pre-SBRT Child–Pugh score		
5–6	79 (92.9%)	73 (85.9%)
7	2 (2.4%)	8 (9.4%)
8–9	4 (4.7%)	4 (4.7%)
Interval (months)		
Median (IQR)	15 (7–24)	
Tumor size (cm)		
Median (IQR)	1.7 (1.5–2.2)	1.7 (1.4–2.2)
Gross tumor volume (cm^3^)		
Median (IQR)	3.6 (2.0–5.9)	2.9 (1.7–4.6)
Planning target volume (cm^3^)		
Median (IQR)	21.0 (15.6–33.2)	20.9 (15.1–27.5)
Normal liver volume (cm^3^)		
Median (IQR)	1210 (1046–1334)	1166 (996–1309)
Mean liver dose (Gy)		
Median (IQR)	5.5 (3.8–6.9)	5.0 (3.8–6.6)
Dose fractionation		
36 Gy/3fx	7 (8.2%)	3 (3.5%)
45 Gy/3fx	60 (70.6%)	63 (74.1%)
48 Gy/4fx	2 (2.4%)	2 (2.4%)
60 Gy/4fx	15 (17.6%)	12 (14.1%)
60 Gy/3fx	1 (1.2%)	2 (2.4%)
Others	0 (0.0%)	3 (3.5%)^†^

*Abbreviations*: SBRT, stereotactic body radiation therapy; IQR, interquartile range; ECOG, Eastern Cooperative Oncology Group.

*Local treatments include transarterial chemoembolization, radiofrequency ablation, percutaneous ethanol injection, and surgical resection. Previous local treatment in the second SBRT session is the value of the interim period between the first and second SBRT.

^†^Other dose fractionations include 40 Gy/4fx, 30 Gy/3fx, and 32 Gy/4fx for patients with Child–Pugh class B.

### Deformable image registration and cumulative liver dose

**Figure 1:**
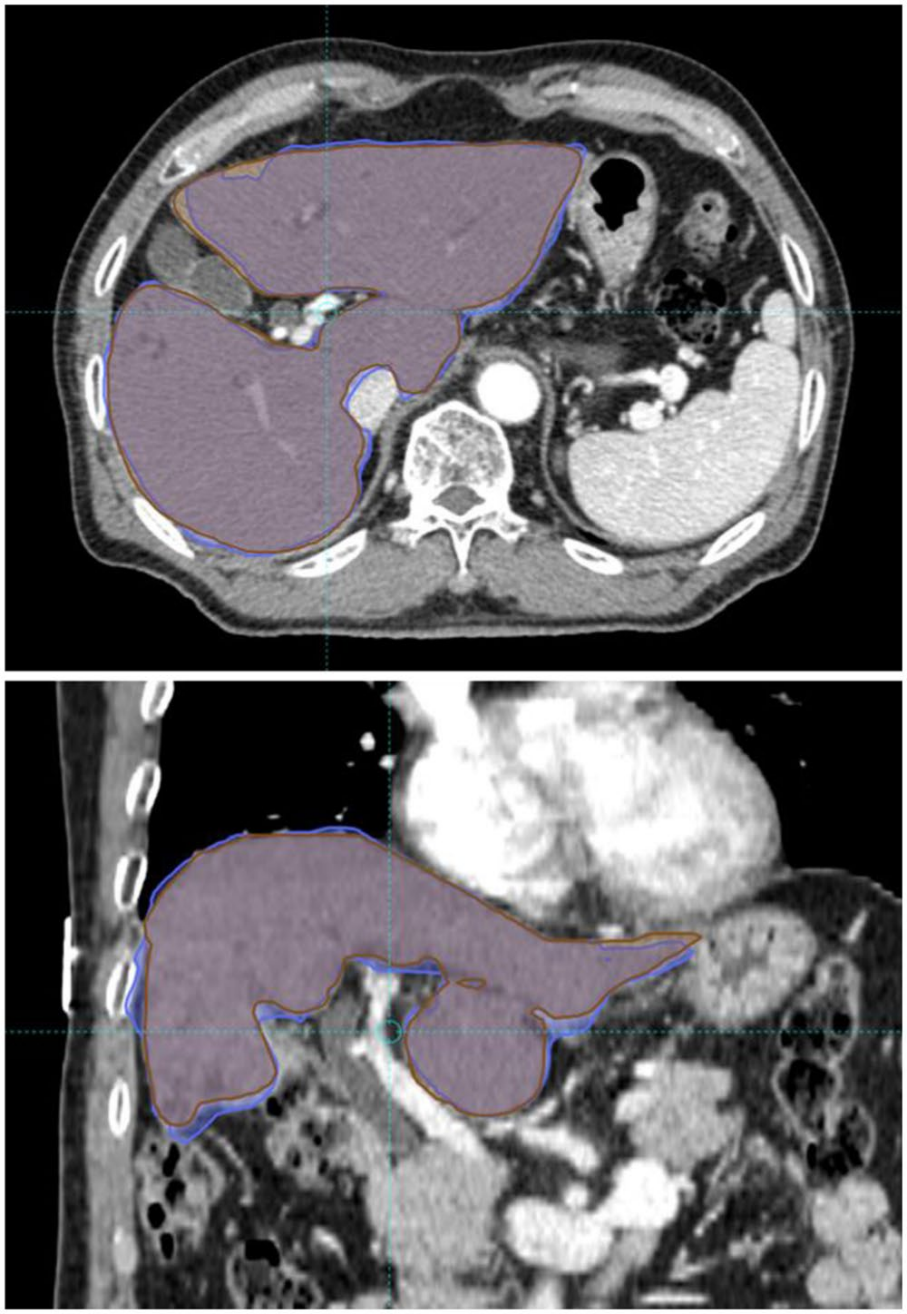
An example of deformable image registration. Liver contours were manually drawn on each computed tomography (CT) simulation image. The liver contour on the first CT simulation was deformed and registered onto the second CT image. The Dice similarity coefficient (DSC) was calculated between manually drawn liver contours in second CT simulation (brown) and the deformed contour from first CT simulation (blue). The DSC value of this patient was 0.94.

**Figure 2:**
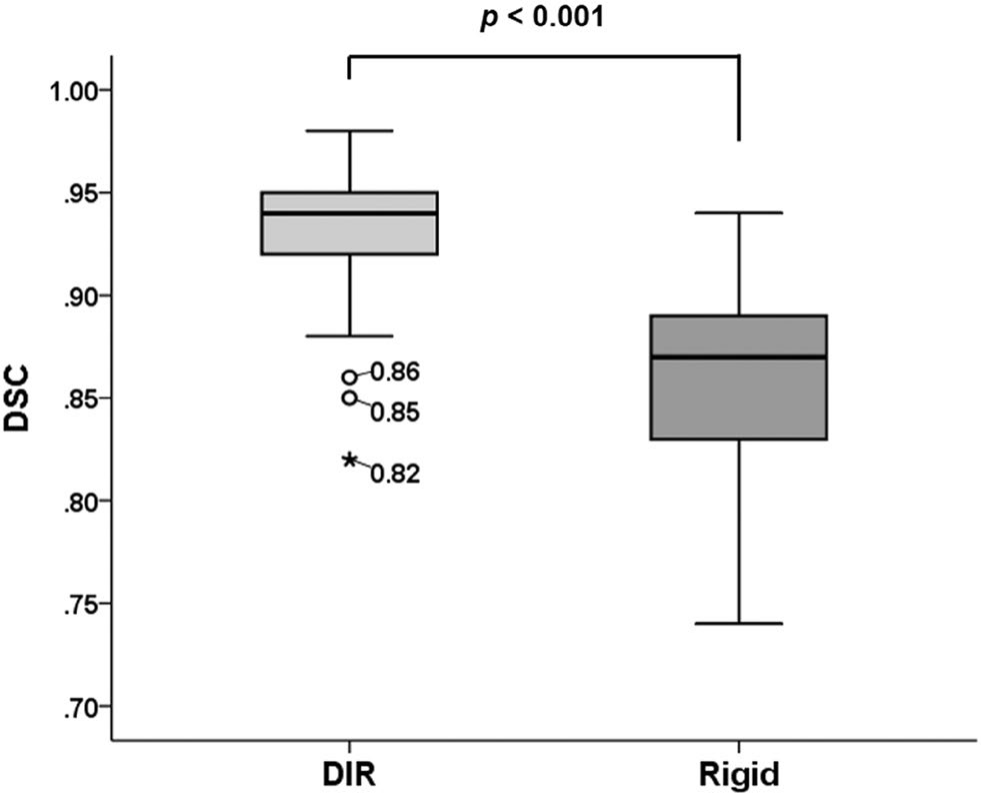
Box plot of the Dice similarity coefficient indices of deformable and rigid image registrations. Outliers that deviated from the boundary of the interquartile range (IQR) by more than 1.5-times the IQR are indicated with circles or asterisks and the corresponding values.

DIR was performed in all patients, and an example DIR is presented in Fig. 1. Mean Dice similarity coefficient (DSC) index value was 0.93 with a standard deviation of 0.03. The DSC index value was >0.9 in 79 (92.9%) registrations, with a value < 0.85 in one registration. The DSC index of rigid registration (single transformation vector for all voxels) was always inferior to that of DIR. DSC indices of DIR and rigid registration are presented in Fig. 2.

**Figure 3:**
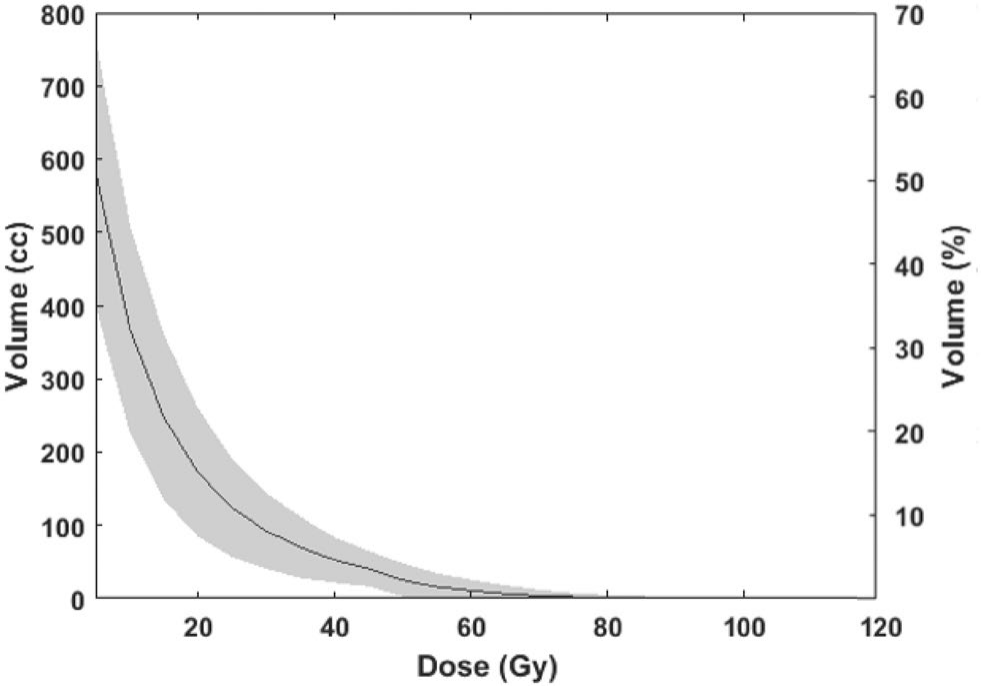
Cumulative dose-volume histogram of normal liver after two sessions of stereotactic body radiation therapy (mean ± standard deviation).

Median cumulative MLD was 9.3 Gy (range, 4.3–19.5; IQR, 7.6–11.7). A cumulative MLD > 13 Gy was received in 10 (11.8%) patients, including a radiation-induced liver disease (RILD) patient who received 19.3 Gy. In addition, 13 (15.3%) patients could not achieve a preserved volume of uninvolved liver irradiated less than 15 Gy exceeding 700 cm^3^ (rV_15Gy_ > 700 cm^3^). The detailed cumulative dose-volume relationship of the liver is shown in Table 2, and the histogram of cumulative dose in mean value with standard deviation is shown in Fig. 3.

**Table 2:** Summary of the cumulative dose–volume parameters.

	V_5Gy_	V_10Gy_	V_15Gy_	V_20Gy_	V_25Gy_	V_30Gy_	V_40Gy_	V_50Gy_
Volume (cm^3^)								
Median	553	339	216	151	108	82	49	22
IQR	456–682	272–447	157–316	107–219	73–156	54–110	32–64	9–35
Volume (%)								
Median	49.8	28.8	19.6	13.8	9.5	7.2	4.1	1.8
IQR	39.1–61.5	22.6–39.2	14.1–27.8	9.7–19.1	6.8–13.8	4.7–10.2	2.5–5.7	0.6–3.1
	**V**_**60Gy**_	**V**_**75Gy**_	**V**_**90Gy**_	**V**_**105Gy**_	**V**_**120Gy**_	**D**_**max**_ **(Gy)**	**rV**_**15Gy**_ **(cm** ^**3**^ **)**	**MLD (Gy)**
Volume (cm^3^)								
Median	4	0	0	0	0	66.4	871	9.3
IQR	0–15	0–1	0–0	0–0	0–0	56.9–79.5	743–1051	7.6–11.6
Volume (%)								
Median	0.3	0	0	0	0		**<700 cm** ^**3**^	**>13 Gy**
IQR	0–1.3	0–0.1	0–0	0–0	0–0		*n* = 13 (15.3%)	*n* = 10 (11.8%)

*Abbreviations*: IQR, interquartile range; rV_15Gy_, reverse V_15Gy_ (normal liver volume irradiated below 15 Gy); D_max_, maximal dose; MLD, mean liver dose.

Volume of liver (cm^3^ or %) irradiated at X Gy (V_XGy_).

### Clinical outcomes

The overall response rate was 74.7% (complete response [CR] in 93 and partial response [PR] in 34 lesions) within 6 months after each SBRT. The tumor response rate was comparable for the two SBRT sessions (76.5% vs. 72.9%, p = 0.708). An example of good response of HCC after repeated SBRT is shown in Supplementary Fig. S1. After a median follow-up time of 45 months (IQR, 31–55), the overall 3-year local control rate was 93.3% in all treatment sessions. The 3-year local control rates of the first and second SBRT were not significantly different (94.9% vs. 90.4%, p = 0.667). Notably, there was no case of re-irradiation for local failure after the first SBRT. The second SBRT was performed for intrahepatic elsewhere recurrence in all patients.

### Hepatic toxicity and liver function

**Figure 4:**
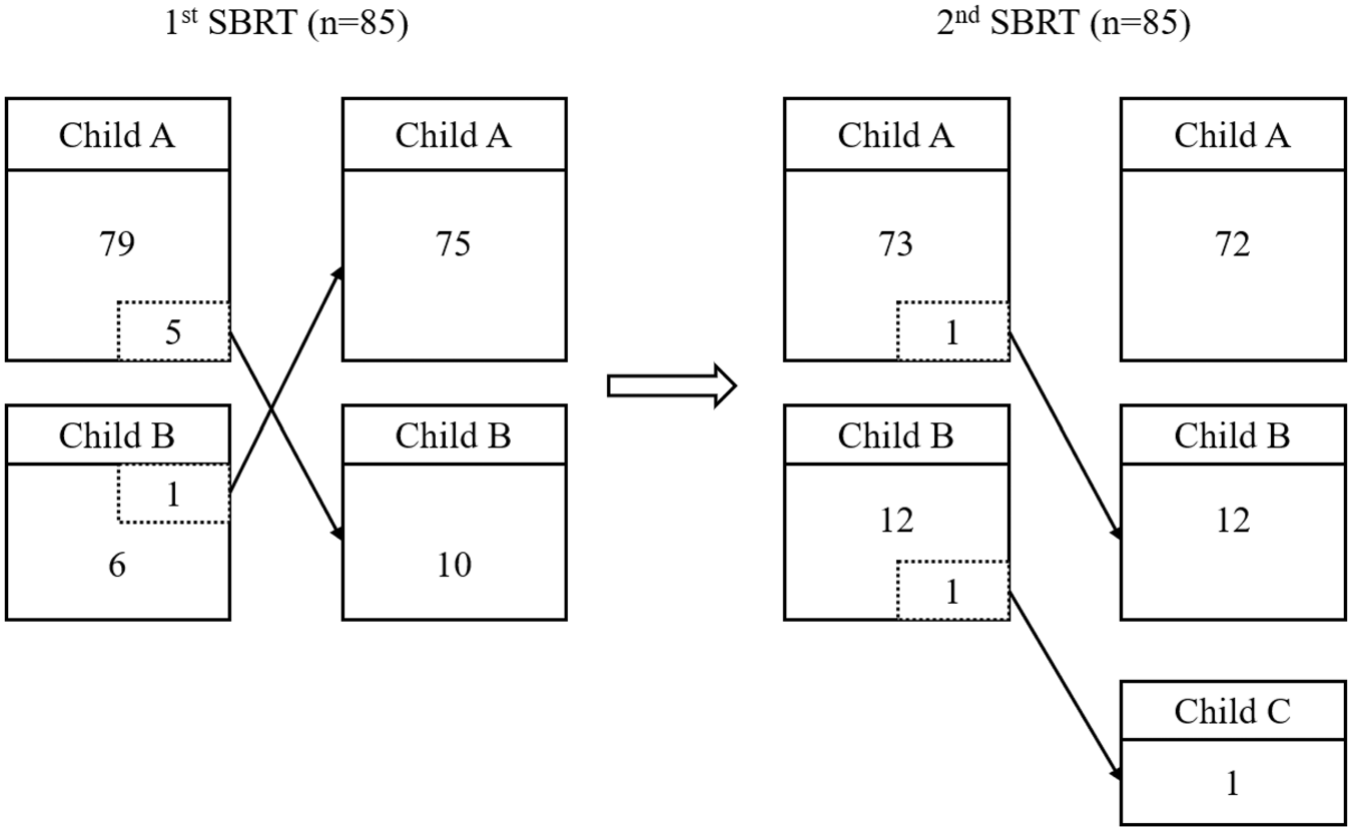
Child-Pugh classification changes in repeated stereotactic body radiation therapy.

Of the 170 SBRT sessions reviewed, only three cases of RILD developed (Table 3). One patient with Child-Pugh class A and sufficient liver volume (1,274 cm^3^) experienced grade 3 aspartate aminotransferase (AST)/alanine aminotransferase (ALT) elevation after the first SBRT of 60 Gy administered in three fractions. It spontaneously resolved within 2 months with supportive care only. He did not experience liver function deterioration or RILD after the second SBRT of 45 Gy administered in three fractions. The other two patients who experienced RILD after their second SBRT of 45 Gy administered in three fractions had Child-Pugh class B (Child-Pugh score 7) hepatic function before the second SBRT. One of these patients, a 70-year-old male did not suffer RILD or any hepatotoxicity after the first SBRT. However, liver function deteriorated rapidly with the development of ascites and hepatic encephalopathy after the second SBRT, and he died within 2 months. His cumulative MLD was 12.5 Gy, and the volume of normal liver was 1139.3 cm^3^. The last patient also experienced an increase in Child-Pugh score by 2 after the second SBRT. His cumulative MLD was 19.3 Gy, and normal liver volume was 794 cm^3^. He was alive during the follow-up period but showed gradual worsening of liver function at the last follow-up. In patients with Child-Pugh A hepatic function before the second SBRT, RILD was not noted regardless of cumulative MLD (range, 4.3–19.5 Gy). A scatter plot of the development of RILD after the second SBRT between the cumulative MLD and Child-Pugh score before the second SBRT is shown in Supplementary Fig. S2. The change of Child-Pugh class of all patients throughout the SBRT course is shown in Fig. 4.

**Table 3:** Summary of cases that developed radiation-induced liver disease after the first and second SBRT.

Case	Timing	Pre-SBRT C–P class (score)	Dose fractionation	Normal liver volume	Cumulative MLD	Hepatotoxicity	Post-SBRT C–P class (score)
67/M	First	A (5)	60 Gy/3fx	1274 cm^3^	5.7 Gy	Gr 3 (AST/ALT)^*^	A (5)
70/M	Second	B (7)	45 Gy/3fx	1139 cm^3^	12.5 Gy	Gr 3 (Bilirubin)	C (12)^*^
58/M	Second	B (7)	45 Gy/3fx	794 cm^3^	19.3 Gy	Gr 2 (Bilirubin)^*^	B (9)^*^

*Abbreviations*: SBRT, stereotactic body radiation therapy; C–P, Child–Pugh; MLD, mean liver dose; Gr, grade; AST, aspartate aminotransferase; ALT, alanine aminotransferase.

^*^Toxicities that meet the pre-defined criteria of radiation-induced liver disease.

### Late hepatic effect after repeated SBRT

There was no grade 3 or higher biliary stricture caused by SBRT during the follow-up periods. There were six (7.1%) cases of mild bile duct dilatation in the correlated area with previous SBRT; however, these did not lead to elevated bilirubin levels or require endoscopic intervention. In serial follow-up computed tomography (CT) images, focal parenchymal change and hepatic atrophy with or without capsular retraction was shown in almost all patients. However, no other specific finding was observed in the overlapped high-dose regions exceeding 60 Gy.

## Discussion

Few studies have evaluated the safety of repeated SBRT thus far. Obstacles such as the application of various treatments due to frequent intrahepatic recurrence, natural deterioration of liver function due to liver cirrhosis, and inaccuracies of the cumulative dose calculation after SBRT make it challenging to evaluate the safety of repeated SBRT. Although the applicability of the DIR tool to the upper abdominal organs has not been well evaluated because of such inaccuracies^21^, the relatively high DSC values presented in this study may have helped to achieve a reliable cumulative dose calculation by DIR. In some cases, low DSC values were due to low contrast between the liver and newly developed ascites or large vessels in the liver contours. Therefore, further research is warranted to improve the registration accuracy.

In the present study, we found that repeated SBRT could be safely performed while maintaining high rates of local tumor control. When performing SBRT for primary liver cancers, Quantitative Analyses of Normal Tissue Effects in the Clinic (QUANTEC) recommends a normal liver volume of >700 cm^3^ to be irradiated <15 Gy for 3–5 fractions, with the MLD not exceeding 13 Gy at three fractions^22^. Even if this single session limitation was directly applied to our case series, the violation of the dose recommendation was only 15.3%. SBRT was performed on relatively small HCCs with a median tumor size of 1.7 cm, and there was minimal liver function deterioration between sessions. Repeated SBRT in such carefully selected patients could be safely performed with an overall incidence of 3.5% RILD. However, the patients with a baseline liver function of Child-Pugh class B and a relatively small volume of normal liver (<800 cm^3^) experienced unrecoverable liver function deterioration after the second session of SBRT. Therefore, care should be taken before recommending repeated SBRT in such clinical conditions.

RILD has been defined differently in many studies, but as a non-classic RILD, liver enzyme elevation or elevation of the Child-Pugh score by ≥2 is a widely used index. In the present study, one patient experienced RILD with liver enzyme elevation after the first SBRT but recovered with supportive care. However, after the second SBRT, the Child-Pugh score elevated by ≥2 in two patients and was not restored, and liver enzyme elevation was not observed. Although the underlying pathology of non-classic RILD is not clearly understood, liver function deterioration with Child-Pugh score elevation may be more significant as a clinical endpoint than liver enzyme elevation.

So far, few studies that have analyzed the results of repeated radiotherapy for recurrent HCC with various fractionation schemes. Lo *et al*.^23^ studied the results of repeated SBRT in 14 HCC patients using CyberKnife with a median dose of 41 Gy (range, 34–60) in the first and 40 Gy (range, 25–50) in the second SBRT. They defined non-classic RILD as correlating with grade 3 or higher toxicity as per Common Terminology Criteria for Adverse Events (CTCAE) version 4.03 and reported that RILD occurred in one (7%) patient after the second SBRT, which resolved with symptomatic management. Seol *et al*.^24^ also reported that tolerable re-irradiation without RILD could be performed in 43 HCC patients. However, these two studies did not show the cumulative radiation dose of repeated radiotherapy using the image registration technique.

Kimura *et al*.^25^ performed repeated SBRT in 24 HCC patients for intrahepatic recurrences with 40 or 48 Gy in four fractions. They reported seven (29%) cases of grade 3 or higher toxicities that included AST/ALT elevation, decreased platelet count, and ascites. These toxicities occurred significantly more in Child-Pugh class B patients. We assumed that, compared with our study, their study had an increased toxicity frequency because of a higher proportion of Child-Pugh class B patients (17% at initial SBRT and 25% at second or beyond, respectively) and a marginally higher cumulative MLD of 13.1 Gy. Oshiro *et al*.^26^ showed the results of 83 patients treated with repeated proton beam therapy using DIR and reported no classic or non-classic RILD with a maximal delivered dose to the liver of 66.7–248.1 GyE. However, as with a single session of SBRT^27^, repeated SBRT in patients with advanced liver cirrhosis seems to exhibit higher hepatic toxicity^28,29^. Therefore, further research is needed to confirm the safety of repeated SBRT in patients with chronic liver disease.

The current study also showed a low risk of late biliary toxicity as well as RILD. Several studies have suggested that high-dose radiotherapy for central lesions may be a risk factor for biliary complications^30,31^. However, there was no grade 3 or higher biliary stricture, and only six patients had grade 1 mild dilation of the bile duct without significant bilirubin elevation or the need for endoscopic intervention. In addition, the irradiated liver showed focal atrophy in almost all patients, a previously well-known image finding^32^. Additional atrophy was observed with repeated SBRT, but other findings, such as distortion of vascular structure or biliary stricture, were not observed in the overlapping high-dose area. These findings suggest that after the progression of focal atrophy due to loss of damaged hepatocytes, the previous high-dose radiation is relatively less influential regarding the safety of repeated SBRT.

The present study has certain limitations. Because this was a retrospective study, the results may have a potential bias and should be interpreted cautiously. In addition, it was difficult to define the reason for hepatic function deterioration after SBRT in patients with a background of liver cirrhosis. In fact, confounding factors, such as other locoregional treatments, worsening of cirrhosis itself, or other hepatotoxic effects, may also be related to these hepatic toxicities. Analysis of inter- and intra-observer variability in liver contours was not performed. The inter-observer variation in liver contours is known to be around 0.96 in DSC, which is acceptably high and reliable^33^. To reduce the uncertainty of inter-observer variation, an intravenous contrast agent was used in each case. Also, both target and liver contours were delineated and confirmed by a radiation oncologist with more than 10 years of experience treating HCC. Thus, we believe that the uncertainty may be acceptable in spite of the limitation. Finally, it was difficult to recommend the maximum tolerable dose level as a guideline for repeated SBRT because of the low RILD incidence. However, to our knowledge, the current study is the largest series evaluating hepatic toxicity after repeated SBRT for HCC and was performed according to a relatively consistent protocol. Moreover, the cumulative dose was reliably calculated using the DIR software, which could aid in clinical judgment.

In conclusion, the DIR method used in the present study provided reliable information on the cumulative dose to the liver. In patients with Child-Pugh class A liver function, repeated SBRT for small recurrent HCC could be safely performed with acceptable hepatic toxicity. The safety of repeated SBRT at a cumulative MLD around or above 13 Gy in patients with Child-Pugh class B hepatic function needs further evaluation.

## Methods

### Patients

Medical records of patients who received repeated SBRT for recurrent HCC between January 2007 and December 2015 at the Asan Medical Center were retrospectively reviewed. The detailed inclusion criteria for SBRT were as described in our previous reports^17,27^. The decision of repeated SBRT was taken by a radiation oncologist with more than 10 years experience in HCC treatment by considering the previous irradiated dose and radiation field. Other local treatments between the SBRT sessions, including surgical resection, TACE, RFA, or ethanol injection, were allowed. This study was approved by the Institutional Review Board of the Asan Medical Center, and informed consent was waived off because of the retrospective nature of the study.

### SBRT procedures

The SBRT procedure at our institution was as described in our previous studies^17,27,34^. At least 1 week before CT simulation, three gold fiducial markers (Standard Gold Soft Tissue Markers; CIVCO Medical Solutions, Kalona, IA) were inserted near the tumor under ultrasonographic guidance^35^. Exceptions were patients having surgical clips or compact iodized oil that were expected to be identified in pretreatment fluoroscopy for image guidance. In all patients, four-dimensional (4D) CT was performed using a 16-slice CT system (GE LightSpeed RT 16; GE Healthcare, Waukesha, WI, USA) in the supine position. The 4D CT images that synchronized with the respiratory data were sorted into 10 CT series according to the respiratory phase (Advantage 4D version 4.2; GE Healthcare). To analyze the patients’ respiratory data, a Real-time Position Management gating system (RPM; Varian Medical Systems, Palo Alto, CA, USA) was used. The GTV was delineated on an end-expiratory phase by referring to the diagnostic liver dynamic CT or magnetic resonance imaging (MRI). For respiratory-gated radiotherapy, the internal target volume was extended from the GTV to include tumor movement from 30% to 70% phase. The PTV margin for setup error was 5 mm. Generally, 45–60 Gy was administered in three or four fractions covering 85–90% PTV. SBRT planning was performed using a three-dimensional radiotherapy planning system (Eclipse; Varian Medical Systems) involving either multiple static conformal beams with energies of 6- or 15-MV photons or volumetric-modulated arc therapy technique using a 10-MV flattening filter-free beam. The actual beam delivery was performed with an image guidance and a respiratory-gated beam delivery technique using On-Board Imager (Varian Medical Systems). Fiducial or surrogate marker-assisted image guidance using cone-beam CT and fluoroscopy was performed before each fraction of SBRT.

### Deformable image registration and cumulative dose calculation

**Figure 5:**
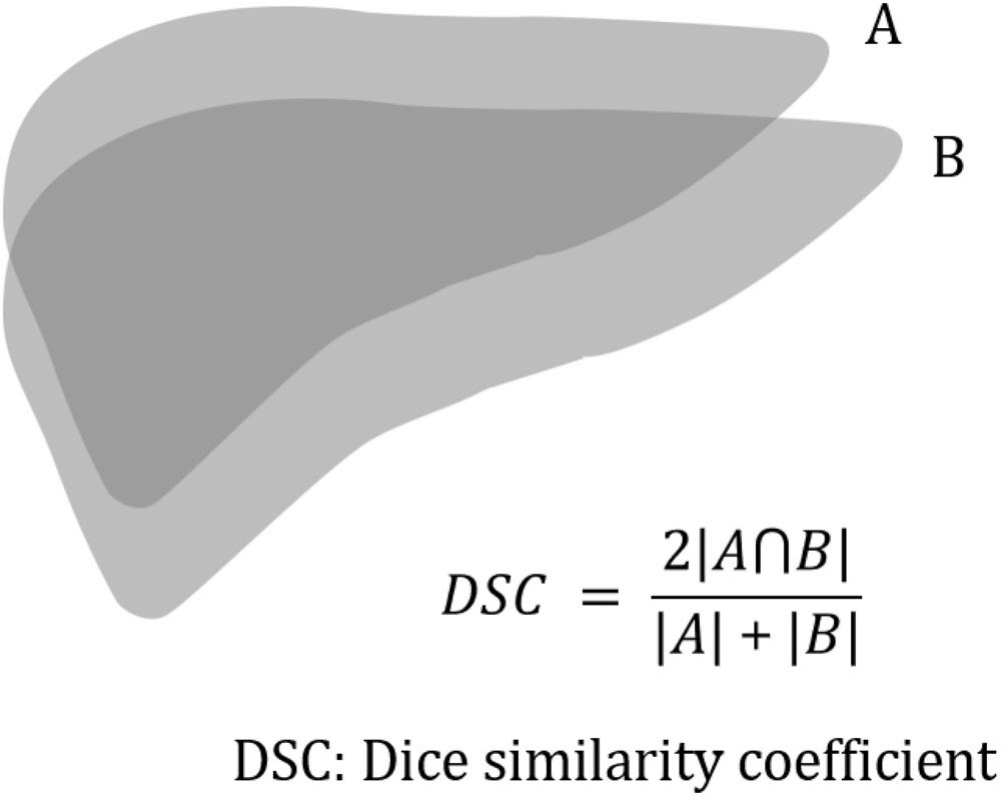
Calculation of the Dice similarity coefficient. The deformed contour of the liver from computed tomography simulation of the first stereotactic body radiation therapy (SBRT) to that of the second SBRT via deformable image registration (**A**) and manually delineated liver contour of the second (**B**) were compared.

CT simulation images and the treatment plan of the first session of SBRT were deformed and registered with the images and plan of the second session to calculate the cumulative hepatic dose. For this, DIR software (Mirada RTx; Mirada Medical Ltd, Oxford, UK), which applies intensity-based voxel to voxel transformation vectors, was used. Liver contours on each simulation CT image were manually drawn by a radiation oncologist. The DSC index between the deformed liver contour on CT images for the first SBRT and the liver contour obtained on the second SBRT was calculated to evaluate DIR performance (Fig. 5).

Cumulative dose-volume parameters of the liver after the first and second SBRT were calculated. Maximal liver doses and MLDs were measured. The volume of the uninvolved liver (liver volume other than GTV) irradiated more or less than the specific cumulative dose (V_5Gy_ to V_120Gy_, and reverse-V_5Gy_, or rV_5Gy_ to rV_120Gy_, 5 Gy increment, respectively) was also evaluated.

### Evaluation of clinical outcome and hepatic toxicity

All patients were evaluated with physical examination, complete blood count, liver function test, tumor markers, and dynamic enhanced CT or MRI within 1 month before SBRT. All patients were evaluated during SBRT to assess acute toxicity with laboratory tests and were followed every 1–3 months after treatment by repeating the evaluations performed prior to SBRT treatment at each visit. SBRT response was evaluated according to the Response Evaluation Criteria in Solid Tumors 1.1. The best response within 6 months after SBRT was determined, and the response rate was defined as CR or PR. Local failure was defined as the recurrence of the treated lesion. RILD was defined when one of the following conditions was satisfied without disease progression within 3 months after SBRT: (1) an increase of ≥2 in the Child-Pugh score or (2) an elevation in AST/ALT or alkaline phosphatase (ALP) of at least 5-fold and/or that of bilirubin of at least 3-fold compared to either the upper normal limit or the pretreatment level corresponding to grade 3 or higher hepatic toxicity according to the CTCAE version 4.03 (https://evs.nci.nih.gov/ftp1/CTCAE/About.html). Late toxicities including biliary toxicity, chronologic liver function changes, and image findings were also evaluated.

### Statistical analysis

The follow-up duration and survival time were measured from the start date of the first SBRT. The Kaplan-Meier method and log-rank test were used for survival analysis. Chi-square, t-test, and logistic regression analysis were used to compare risks of parameters. P < 0.05 was considered statistically significant. SPSS Statistics version 21 (IBM SPSS Statistics, Armonk, NY) was used for all statistical analyses.

## Electronic supplementary material

Supplementary Figure 1, Figure 2.

Dataset 1

## Data Availability

All relevant data are within the paper and its Supplementary Dataset File. File name: supplementary_dataset_re-SBRT_85patients.xlsx.
