# Supplementary material for: Evaluation of Hepatic Toxicity after Repeated Stereotactic Body Radiation Therapy for Recurrent Hepatocellular Carcinoma using Deformable Image Registration
Source: Sci Rep. 2018 Nov 1;8:16224. doi: 10.1038/s41598-018-34676-1 (PMC6212421; doi:10.1038/s41598-018-34676-1)

Supplementary Materials

Evaluation of Hepatic Toxicity after Repeated Stereotactic Body Radiation Therapy for Recurrent Hepatocellular Carcinoma using Deformable Image Registration

Sumin Lee, Hojin Kim, Yunseo Ji, Byungchul Cho, Su Ssan Kim, Jinhong Jung, Jungwon Kwak, Jin-hong Park, Sang-wook Lee, Jong Hoon Kim, Sang Min Yoon*

Supplementary Figure S1. An example of good response of hepatocellular carcinoma after repeated stereotactic body radiation therapy.

Supplementary Figure S2. A scatter plot of the development of radiation-induced liver disease after the second stereotactic body radiation therapy (SBRT) between the cumulative mean liver dose and Child-Pugh score before the second SBRT.

Supplementary Figure S1. An example of good response of hepatocellular carcinoma after repeated stereotactic body radiation therapy.

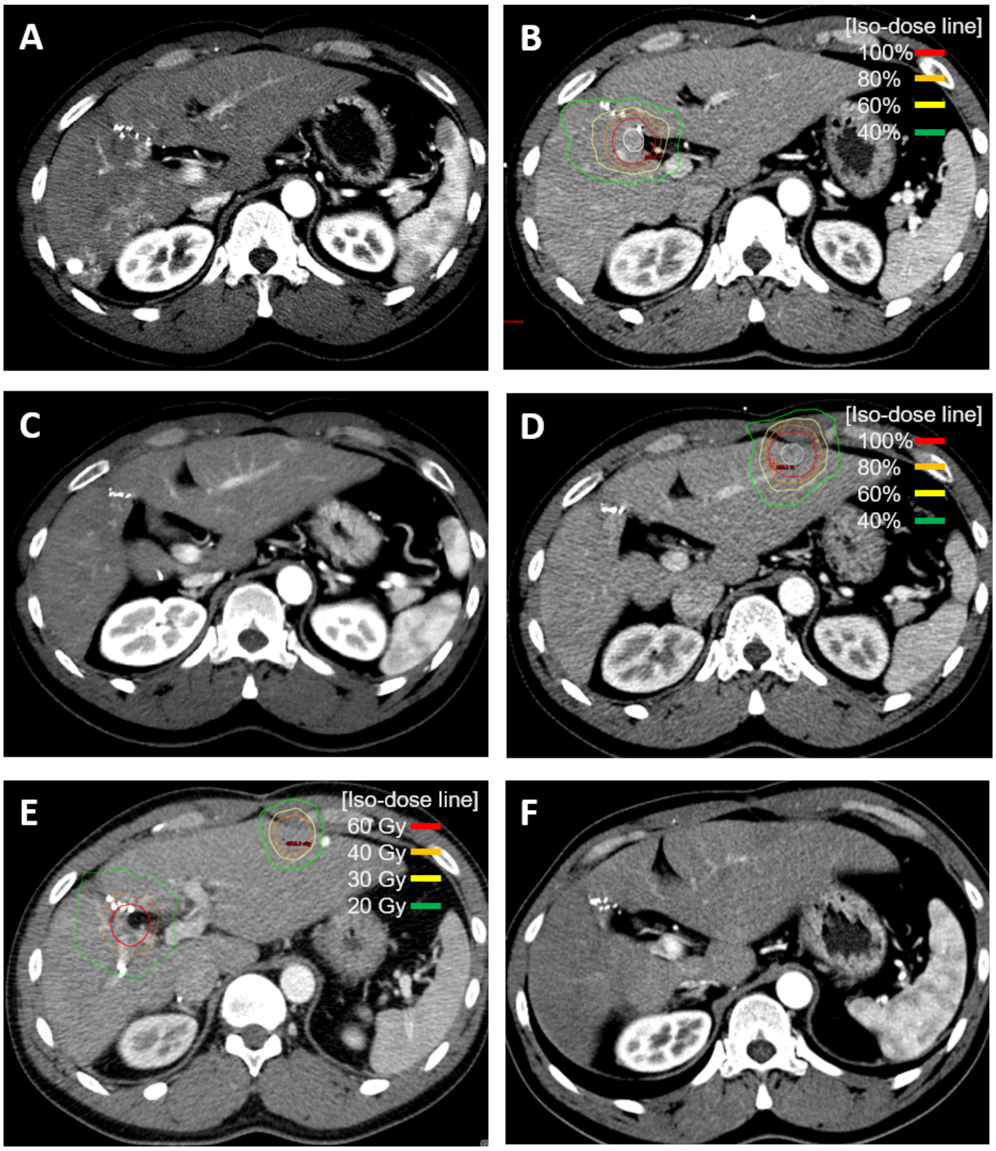

The patient received right anterior segmentectomy for HCC. Two years later, HCC recurred in hepatic segment VII around the previous hepatic resection margin (A: arterial phase). He received first SBRT of 60 Gy in four fractions to the HCC in segment VII (B: planning CT and iso-dose lines). Two years after the first SBRT, the HCC in segment VII did not recur on follow-up CT images, but another recurred HCC was shown in segment III (C: arterial phase). Later, he received second SBRT of 45 Gy in three fractions to the recurred HCC in segment III (D: planning CT and iso-dose lines, E: Combined plan between the first and second SBRT). Six months after the second SBRT, HCC in segment III also disappeared on follow-up CT images and there was no evidence of recurrent HCC (F: arterial phase).

Supplementary Figure S2. A scatter plot of the development of radiation-induced liver disease after the second stereotactic body radiation therapy (SBRT) between the cumulative mean liver dose and Child-Pugh score before the second SBRT.

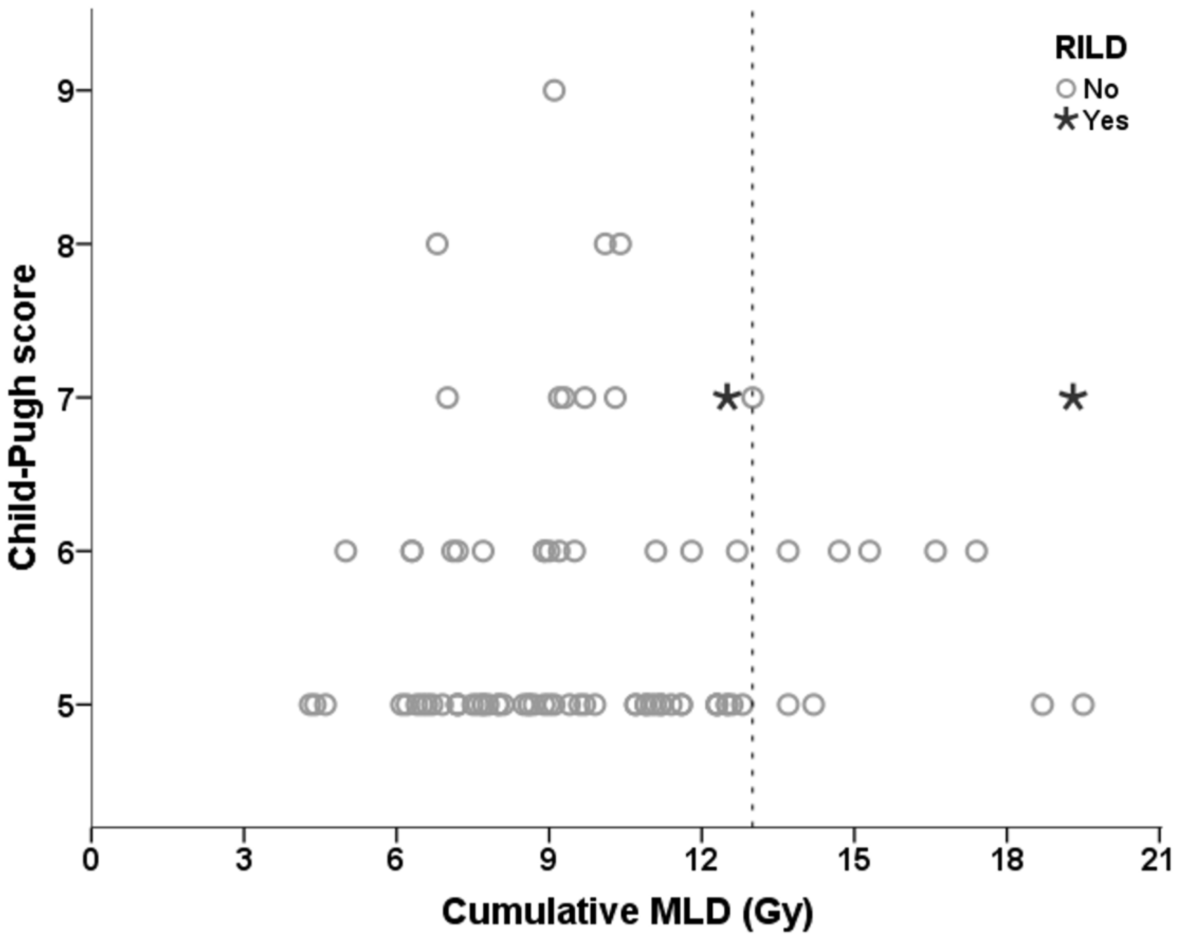

Supplement: Supplementary file 1 — Supplementary Figure 1, Figure 2. [file 41598_2018_34676_MOESM1_ESM.pdf]
